# Metabolism of Zearalenone in the Course of Beer Fermentation

**DOI:** 10.3390/toxins3020134

**Published:** 2011-02-14

**Authors:** Kohei Mizutani, Yasushi Nagatomi, Naoki Mochizuki

**Affiliations:** Asahi Breweries, Ltd., Analytical Technology Laboratory, 1-1-21, Midori, Moriya, Ibaraki, Japan; Email: yasushi.nagatomi@asahibeer.co.jp (Y.N.); naoki.mochizuki@asahibeer.co.jp (N.M.)

**Keywords:** zearalenone, metabolite, beer, fermentation, liquid chromatography/tandem mass spectrometry (LC/MS/MS)

## Abstract

Zearalenone (ZON) is a mycotoxin with estrogenic activity, produced by members of *Fusarium* species, and is found worldwide in a number of cereal crops. It is known to have four active metabolites (α-zearalenol (α-ZOL), β-zearalenol (β-ZOL), α-zearalanol (α-ZAL), and β-zearalanol (β-ZAL)). A highly sensitive analytical method using liquid chromatography/tandem mass spectrometry using electrospray ionization (LC-ESI-MS/MS) has been established and validated in order to analyze ZON and its metabolites in beer and malt samples. The metabolism of ZON in the course of beer fermentation was further characterized using the artificially contaminated wort by this established method. In the fermented sample, 85.9% of ZON was converted to β-ZOL, which has lower estrogenic activity than that of ZON. These findings indicate that the health risk to humans due to ZON in beer is reduced during the fermentation process.

## 1. Introduction

**Figure 1:**
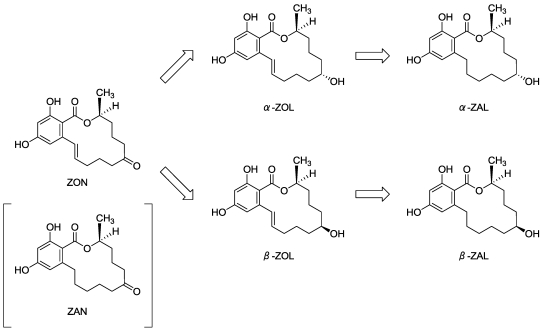
Chemical structures of Zearalenone (ZON), its major metabolites and internal standard used.

Zearalenone (ZON) is a mycotoxin produced by *Fusarium* species (e.g., *F. graminearum*, *F. crookellense*, and *F. acuminatum*) and occurs in several grains including maize, barley, oats, wheat, and sorghum [1,2]. ZON has the ability to bind to estrogen receptors (ERs) and induces estrogenic syndromes including uterine enlargement, swelling of the vulva and mammary glands, and pseudopregnancy through the intake of contaminated grains [3]. For this reason, risks of ZON contamination is of international concern, and has become a substantial problem in beer production [4,5,6]. One study has reported that ZON is converted to its metabolites, α-zearalenol (α-ZOL) and β-zearalenol (β-ZOL), which are further converted to α-zearalanol (α-ZAL) and β-zearalanol (β-ZAL), respectively (Figure 1) [7,8]. Among these metabolites, α-ZOL, α-ZAL, and β-ZAL have relatively higher estrogenic activity than that of ZON (their binding affinities for ER rank: α-ZOL > α-ZAL > β-ZAL > ZON > β-ZOL [9,10]. Meanwhile, it was indicated that yeast metabolizes ZON to β-ZOL rather than α-ZOL [11]. However, there have been no previous studies during fermentation on zearalanols (ZAL) having higher estrogenic activity than that of ZON. Therefore, to help in predicting the human risk due to contamination in brewing by not only ZON but its multiple metabolites, we developed and validated an analytical method for simultaneously determining these metabolites in barley malt, the main raw material of beer, and in beer during the fermentation process. Then, we performed a laboratory-scaled brewing experiment using the wort artificially contaminated with ZONs and closely elucidated the fates of ZON and of its metabolites by the developed analytical method.

## 2. Experimental

### 2.1. Materials

ZONs standards (ZON, α-ZOL, β-ZOL, α-ZAL and β-ZAL) and the internal standard, Zearalanone (ZAN), were purchased from Sigma Aldrich (Germany). Acetonitrile (HPLC grade), methanol (HPLC grade) and ammonium acetate (analytical grade) for the mobile phase, and methanol (analytical grade) for sample preparation were from Kanto Chemical (Japan). Potassium chloride (analytical grade) was from Wako Chemical (Japan). The SPE cartridge, Supelclean ENVI-18 6 ml (1 g) was from Supelco (USA). The glass microfiber filter GF/A was from Whatman (UK). The PTFE filter (pore size 0.2 μm) was from Toyo Roshi kaisha (Japan). The control two-raw malt was from Canada Malting (Canada) and the control beer was from Asahi Breweries (Japan), which were used for the method’s validation test.

### 2.2. LC/MS/MS Conditions

The analysis was performed with an Alliance 2960 system (Waters, USA) for liquid chromatography and a Quattro Ultima triple stage quadrupole instrument equipped with an ESI interface (Waters) for mass spectrometry. The chromatographic separation was achieved on Nova-Pak phenyl column (150 × 2.0 mm i.d., 4 μm, Waters) at 40 °C, using a linear gradient program. Solvents A, B, and C were methanol, water, and 10 mM ammonium acetate, respectively. Elution was carried out at a flow rate of 0.2 mL applying the following gradient program: 0 min 20% A; 1 min 20% A; 20 min 60% A; 30 min 60% A; 31 min 20% A, and 5% C constant. The injection volume was 20 μL. For the mass spectrometry, the following settings were used in a negative ionization mode: capillary voltage 2.5 kV; cone voltage 90 V; source temperature 120 °C; desolvation temperature 300 °C. Table 1 shows the settings for a multiple reaction monitoring (MRM) mode. 

**Table 1:** Liquid chromatography/tandem mass spectrometry (LC/MS/MS) conditions for fragmentations.

ZONs	Precursor ions (m/z)	Product Ions (m/z)	
		For Quantification m/z	For Qualification m/z
		(Collision Energy, eV)	(Collision Energy, eV)
ZON	317	131(28)	175(18), 273(15)
α-ZOL	319	174(22)	160(28), 275(18)
β-ZOL	319	275(18)	160(24), 174(26)
α-ZAL	321	277(22)	303(18), 259(20)
β-ZAL	321	277(22)	303(20), 259(22)
ZAN (I.S.)	319	275(18)	205(20)

### 2.3. Sample Preparation

The malt sample was prepared with reference to a previously described method [12]. Twenty-five grams of finely ground malt with 2.5 g of potassium chloride added was extracted with 100 mL of acetonitrile-water (75:25, v/v) by mixing with a magnetic stirrer for 15 minutes. After filtration through a glass microfiber filter, 10 mL of the extract was diluted with 90 mL water and was adjusted to pH 4.0 with acetic acid. 40 mL of this solution was applied to the SPE cartridge, Supelclean ENVI-18 (1 mg), preconditioned with 5 mL of methanol and 5 mL of water. After the passage of the sample solution through the cartridge, 5 mL of water was used for washing the cartridge. Then, ZONs were eluted with 5 mL methanol. The eluate was evaporated under a stream of nitrogen and the residue was well redissolved in 3 mL of methanol-water (75:25, v/v). After filtration through the PTFE filter, the sample solution was analyzed by LC/MS/MS. For the analysis of beer, samples were degassed by sonication for 45 minutes and a volume of 50 mL was applied directly to the SPE cartridge. The subsequent steps were carried out as described for the aforementioned malt analysis. For accurate quantification, the internal standard (ZAN) [12,13] was added before the sample preparation at a concentration of 10 ng/g. The standards of ZON and its metabolites were added to samples artificially contaminated with them before the sample preparation at levels of 2, 5, 10, 20, 50, 100, 200 ng/g (ppb) for the validation test and to have the calibration curve.

### 2.4. Fermentation Test

To characterize the metabolism of ZON in brewing fermentation, small-scale fermentation testing was conducted in our laboratory. Two liters of wort produced from uncontaminated malt was spiked with ZON at a concentration of 100 ng/g and was fermented at 12 °C for 18 days with 10 g of brewers’ yeast (*Saccharomyces pastrianus*), which is currently used for the production in our brewery. *S. pastrianus* is classified as a bottom-fermented yeast, which can sink to the bottom of the wort after fermentation. Meanwhile, another type of yeast used in beer production, *S. cerevisia*, is a top fermented yeast. Fourteen sampling points in the 18-day trial period were established as shown in Table 2. ZON and its metabolites’ levels were determined at each point, based on the beer analysis procedure in Section 2.2: “*LC/MS/MS conditions*” and Section 2.3: “*Sample preparation*”. 

**Table 2:** Temporal changes in concentration of ZON and its metabolites.

Fermentation Time	Day:							1	2	3	4	7	10	14	18
	Hour:	0	1	2	4	6	8	24	48	72	96	168	240	336	432
ZON	ng/g	100	82.4	78.7	77.5	73.0	69.1	62.7	52.8	45.4	37.4	22.8	18.1	13.4	9.3
α-ZOL		0	－	－	－	－	－	－	－	－	2.5	2.0	4.9	2.5	2.0
β-ZOL		0	9.2	14.1	14.7	16.9	19.7	25.0	35.6	42.3	51.2	63.9	79.0	84.3	85.9
α-ZAL		0	－	－	－	－	－	－	－	－	－	－	－	－	－
β-ZAL		0	－	－	－	－	－	－	－	－	－	－	－	－	－
Total ZONs	ng/g	100	91.6	92.8	92.2	89.9	88.8	87.7	88.4	87.7	91.1	88.7	102	100.2	97.2

## 3. Results and Discussion

### 3.1. LC/MS/MS Conditions

**Figure 2:**
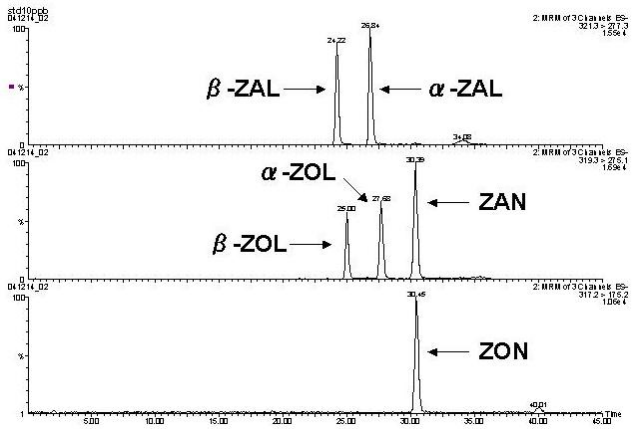
Chromatograms of standard for ZON and its metabolites at a concentration of 10 ng/g.

Consistent with the findings of previous studies [14], ZON and its metabolites showed higher sensitivity in the negative ionization mode when the tuning of MS/MS conditions were performed. Thus, the deprotonated molecule [M − H]^−^ was selected for each precursor ion. The product ion, most abundantly found in the fragmentation spectra of the standard, was selected as the quantification ion. The product ions second and third in abundance were also selected as qualification ions (Table 1). Nova-Pak phenyl column showed a good performance in separating ZONs on the chromatogram because ZONs naturally have benzene rings in their structures (Figure 2). The π-π interaction of the benzene ring between the phenyl column and ZONs is likely to be a contributing factor of the sharpened and well separated peak of ZONs.

### 3.2. Validation Tests

Validation tests were performed for malt and beer. Table 3 shows the results for the malt analysis and Table 4 for the beer analysis. Good recoveries of 101.1%–119.7% were obtained with the spiked samples at a concentration of 10 ng/g for ZONs, with sufficient repeatability of RSD (repeatability standard deviation) = 2.2%–8.1% (n = 5). The calibration curves obtained with the spiked samples were linear at levels of 2, 5, 10, 20, 50, 100, 200 ng/g (ppb), showing correlation coefficients (*r*) of 0.9997–1.0000. The lowest level of LOQ (limit of quantification) in ZON and its metabolites was 2 ng/g (ppb) for beer and malt, which was the minimum point of the analytical line.

**Table 3:** Recovery, repeatability (RSD), and linearity (correlation coefficient, *r*) for determination of ZON and its metabolites in malt.

ZONs	ZON	α-ZOL	β-ZOL	α-ZAL	β-ZAL
Recovery (%)	110.4	111.9	115.8	113.9	119.7
10 ng/g, n = 5					
RSD (%)	4.0	3.0	5.4	4.3	8.1
10 ng/g, n = 5					
R	0.9999	0.9999	0.9999	0.9998	0.9997
2–200 ng/g					

**Table 4:** Recovery, repeatability (RSD), and linearity (correlation coefficient, *r*) for the determination of ZON and its metabolites in beer.

ZONs	ZON	α-ZOL	β-ZOL	α-ZAL	β-ZAL
Recovery (%)	102.3	104.8	101.1	111.3	102.8
10 ng/g, n = 5					
RSD (%)	2.2	3.4	7.9	7.2	5.1
10 ng/g, n = 5					
R	1.0000	1.0000	1.0000	1.0000	1.0000
2–200 ng/g					

### 3.3. Fermentation Test

**Figure 3:**
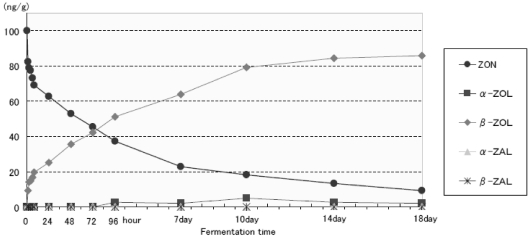
Line chart visualizing temporal changes in concentration of ZON and its metabolites over the fermentation period.

Table 2 shows temporal changes in concentration of ZON and its metabolites during the small-scale fermentation test using commercial wort and brewers’ yeast (*Saccharomyces pastrianus*). The concentration of ZON decreased from 100ng/g to 9.3 ng/g in 18 days. On the other hand, the concentration of β-ZOL, a ZON metabolite, increased throughout the fermentation process and eventually reached up to 85.9 ng/g in the last day of the trial. A slight amount of α-ZOL, an isomer of β-ZOL, was also detected from day 4 onward, reaching 2.0 ng/g in day 18 in the trial period. Furthermore, α-ZAL or β-ZAL was not detected in this fermentation trial. Figure 3 also shows that the level of β-ZOL exceeded that of ZON, 96 hours after the trial started. These results suggest that ZON is mainly metabolized to β-ZOL during brewing fermentation and that β-ZOL is not further metabolized to β-ZAL by brewers’ yeast unlike the way it is metabolized in the liver [7,8]. The reason for this is likely that a carbonyl group is reduced by brewers’ yeast (Figure 1) [15]. On the other hand, reduction of the double bond to a single bond was not observed when the reduction is made inside the liver.

According to previous reports, β-ZOL has an approximately 10 to 20-fold weaker binding affinity for ER than ZON and some 100-fold weaker than α-ZOL [9,16]. Increase in the concentration of β-ZOL suggests that the health risk posed by ZON contamination in beer is maintained at an extremely low level because β-ZOL exhibits lower estrogenic activity than ZON does.

## 4. Conclusions

With respect to ZON contamination risks in brewing fermentation, it is important to monitor not only ZON but also its metabolites due to the differences in their binding affinity for estrogen receptors. Using LC/MS/MS, we developed a novel and sensitive multiple method to simultaneously quantify ZON and its four metabolites, including ZAL, in beer and malt samples. The lowest level of limit of quantification was 2 ng/g (ppb). The laboratory-scaled fermentation test using this method demonstrated that ZON was largely metabolized to β-ZOL (18 days after: ZON 9.3%, β-ZOL 85.9%), which exhibits the lowest estrogenic activity among ZON metabolites. Meanwhile, it was not at all metabolized to α-ZAL or β-ZAL, having the higher estrogenic activity. These findings indicate that ZON risk in beer is reduced during the fermentation process and that the new approach using LC-MS/MS enabled the simultaneous analysis of ZON and its derivatives, having different estrogenic activities, in beer and malt.
